# Crystal structures and Hirshfeld surface analyses of (*N*-hexyl-*N*-methyl­dithio­carbamato-κ^2^ *S*,*S*′)triphenyl­tin(IV) and [*N*-methyl-*N*-(2-phenyl­ethyl)­dithio­carbamato-κ^2^ *S*,*S*′]tri­phenyl­tin(IV)

**DOI:** 10.1107/S2056989018005133

**Published:** 2018-04-06

**Authors:** Rapidah Mohamad, Normah Awang, Nurul Farahana Kamaludin, Mukesh M. Jotani, Edward R. T. Tiekink

**Affiliations:** a Biomedical Science Programme, Faculty of Health Sciences, Universiti Kebangsaan Malaysia, Jalan Raja Muda Abdul Aziz, 50300 Kuala Lumpur, Malaysia; b Environmental Health and Industrial Safety Programme, Faculty of Health Sciences, Universiti Kebangsaan Malaysia, Jalan Raja Muda Abdul Aziz, 50300 Kuala Lumpur, Malaysia; c Department of Physics, Bhavan’s Sheth R. A. College of Science, Ahmedabad, Gujarat 380001, India; d Research Centre for Crystalline Materials, School of Science and Technology, Sunway University, 47500 Bandar Sunway, Selangor Darul Ehsan, Malaysia

**Keywords:** crystal structure, organotin, di­thio­carbamate, Hirshfeld surface analysis

## Abstract

The crystal and mol­ecular structures of two tri­phenyl­tin di­thio­carbamate compounds, *viz*. [Sn(C_6_H_5_)_3_(C_8_H_16_NS_2_)], (I), and [Sn(C_6_H_5_)_3_(C_10_H_12_NS_2_)], (II), are described. The di­thio­carbamate ligand in each mol­ecule coordinates in an asymmetric fashion resulting in heavily distorted tetra­hedral C_3_S coordin­ation geometries for the Sn atoms, with the distortions traced to the close approach of the non-coordinating thione-S atom. The mol­ecular packing in both compounds features C—H⋯π(Sn-phen­yl) inter­actions. In (I), the donors are Sn-phenyl-C—H groups leading to centrosymmetric aggregates, while in (II), the donors are both Sn-phenyl-C—H and methyl-C—H groups leading to supra­molecular chains propagating along the *b* axis. The identified aggregates assemble into their respective crystals with no directional inter­actions between them. An analysis of the Hirshfeld surfaces show distinctive patterns, but an overwhelming predominance (>99% in each case) of H⋯H, C⋯H/H⋯C and S⋯H/H⋯S contacts on the respective Hirshfeld surface.

## Abstract 2

The metal coordination geometry in each of the title mol­ecules, [Sn(C_6_H_5_)_3_(C_8_H_16_NS_2_)] (I) and [Sn(C_6_H_5_)_3_(C_10_H_12_NOS_2_)] (II), is based on a heavily distorted tetra­hedron owing to the asymmetric mode of coordination of the di­thio­carbamate ligand. The persence of C—H⋯π(phen­yl) inter­actions in the crystals lead to dimeric aggregates in (I) and supra­molecular chains (II).

## Chemical context   

A vast array of different di­thio­carbamate anions, ^−^S_2_CN*RR*′, has been prepared, which stems simply from the ability to alter the substituents in the starting amines used to prepare them. A key inter­est in di­thio­carbamate compounds of both transition metals and main-group elements relates to their biological activity (Hogarth, 2012 ▸). Of particular relevance to the present study is the anti-microbial potential exhibited by organotin di­thio­carbamates (Tiekink, 2008 ▸). In an on-going study of biological potential, organotin(IV) species have been complexed with two non-symmetric di­thio­carbamate ligands, namely, with *R* = Me and *R*′ = *n*-Hex and CH_2_CH_2_Ph. Previously, similar species, *i.e. R* = benzyl and *R*′ = CH_2_CH_2_Ph (Mohamad, Awang, Kamaludin & Abu Bakar, 2016 ▸; Segovia *et al.*, 2002 ▸) and *R* = Me and *R*′ = *n*-Bu (Segovia *et al.* 2002 ▸) have been tested for their toxicity using a bioassay based on the inhibition of the growth of *Escherichia coli* with the latter compound being most toxic according to the EC_50_ value measured *in vitro* (Segovia *et al.*, 2002 ▸). These results gave rise to the suggestion that increasing the length of the alkyl chain leads to enhanced solubility/activity of the compound (Segovia *et al.*, 2002 ▸). Complementing these biological investigations (Mohamad, Awang, Kamaludin & Abu Bakar, 2016 ▸; Mohamad, Awang & Kamaludin, 2016 ▸) are structural studies of organotin di­thio­carbamates (Mohamad, Awang, Jotani & Tiekink, 2016 ▸; Mohamad, Awang, Kamaludin, Jotani *et al.*, 2016 ▸; Mohamad *et al.*, 2017 ▸, 2018 ▸) and in continuation of the latter, herein, the crystal and mol­ecular structures of (C_6_H_5_)_3_Sn[S_2_CN(Me)Hex] (I) and (C_6_H_5_)_3_Sn[S_2_CN(CH_3_)CH_2_CH_2_Ph] (II) are reported along with a Hirshfeld surface analysis to provide more details on the mol­ecular packing.
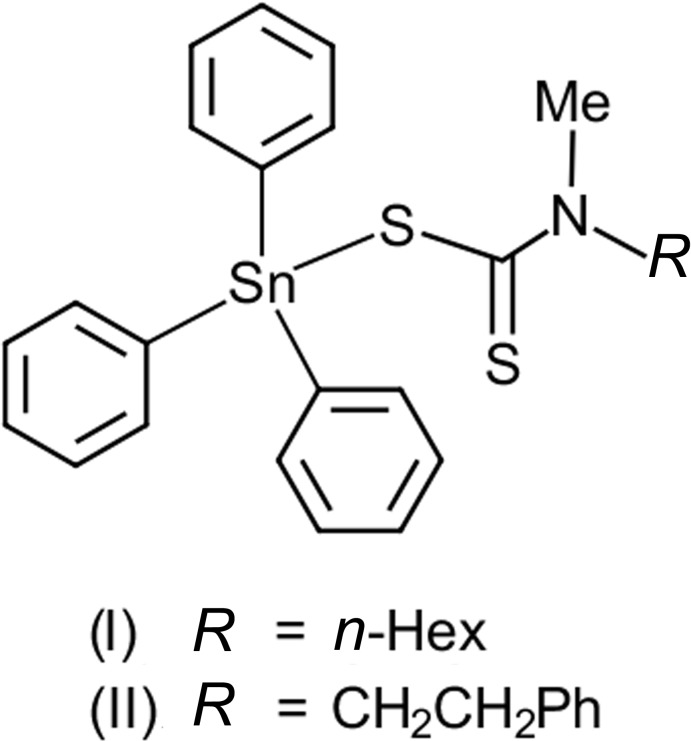


## Structural commentary   

**Figure 1:**
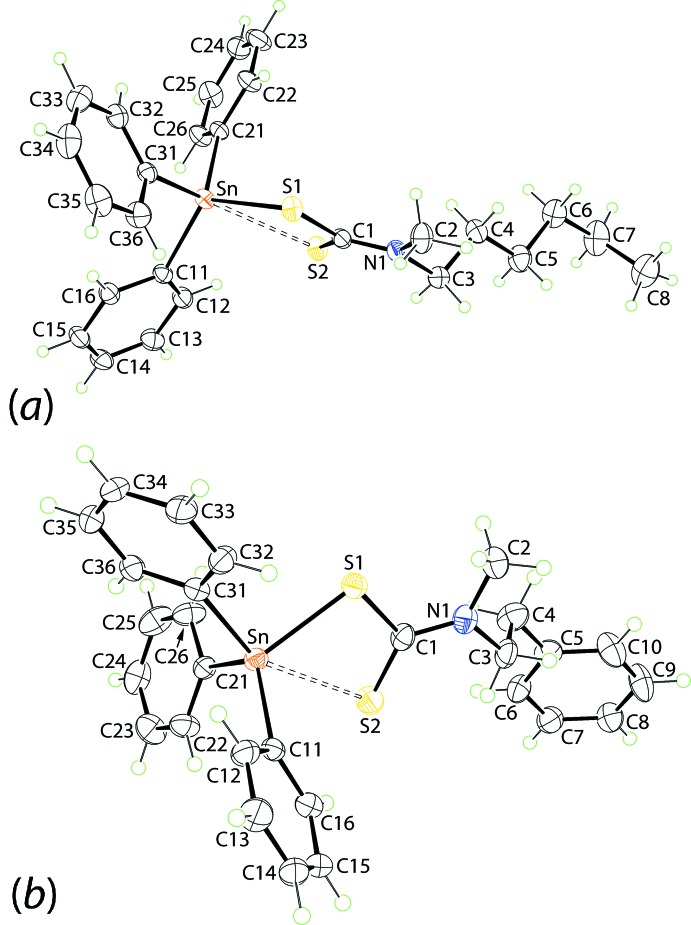
The mol­ecular structures of (*a*) (I) and (*b*) (II), showing the atom-labelling schemes and displacement ellipsoids at the 50% probability level.

The tin atom in (I), Fig. 1 ▸ *a*, is coordinated by three *ipso*-carbon atoms along with a di­thio­carbamate ligand. As seen from Table 1 ▸, the di­thio­carbamate ligand forms quite disparate Sn—S1, S2 bond lengths, with Δ(Sn—S) = (Sn—S_long_ – Sn—S_short_) being 0.64 Å. This asymmetry is confirmed in the differences in the C—S bond lengths with the C1—S1 bond associated with the short Sn—S1 contact, at 1.761 (4) Å, being significantly longer than the C1—S2 bond, *i.e*. 1.688 (4) Å, involving the weakly bound S2 atom. If the S2 atom is ignored, the coordination geometry about the tin atom is distorted C_3_S tetra­hedral with the range of angles being 90.00 (11)°, for S1—Sn—C31, to 121.53 (10)°, for S1—Sn—C11. The wide angle clearly reflects the influence of the close approach of the S2 atom, Fig. 1 ▸ *a* and Table 1 ▸. If the S2 atom is considered a significant bonding inter­action, the resultant C_3_S_2_ donor set is almost perfectly inter­mediate between ideal square-pyramidal (SP) and trigonal–bipyramidal (TP). This is qu­anti­fied in the value of τ = 0.52, which compares with the ideal values for SP and TP geometries of τ = 0.0 and 1.0, respectively (Addison *et al.*, 1984 ▸). In the latter description, the range of angles is wide with the S1—Sn—S2 chelate angle being acute [63.26 (3)°] and with the widest angle [152.54 (11)°] being for S2—Sn—C31. The *n*-hexyl chain is linear up to the terminal methyl group. Thus, the N1—C3—C4—C5, C3—C4—C5—C6 and C4—C5—C6—C7 torsion angles of 175.9 (4), 178.5 (4) and 178.9 (5)°, respectively, indicate + anti-periplanar descriptors but, that of C5—C6—C7—C8, *i.e*. −66.4 (8)°, indicative of a − syn-clinal disposition.

The mol­ecular structure of (II), Fig. 1 ▸ *b*, resembles closely that described for (I). Indeed, a comparison of the key bond lengths included in Table 1 ▸ show there are no chemically significant differences between the common parts of the mol­ecules. In terms of bond angles, for a tetra­hedral description, the range of angles in (II) is smaller, by 2°, than in (I), again, not chemically significant. If the five-coordinate C_3_S_2_ description pertains, the value of τ = 0.60 indicates a distortion towards TP. The phenyl­ethyl chain is kinked as seen in the N1—C3—C4—C5 and C3—C4—C5—C6 torsion angles of −175.8 (3) and 91.9 (5)°, respectively.

## Supra­molecular features   

**Figure 2:**
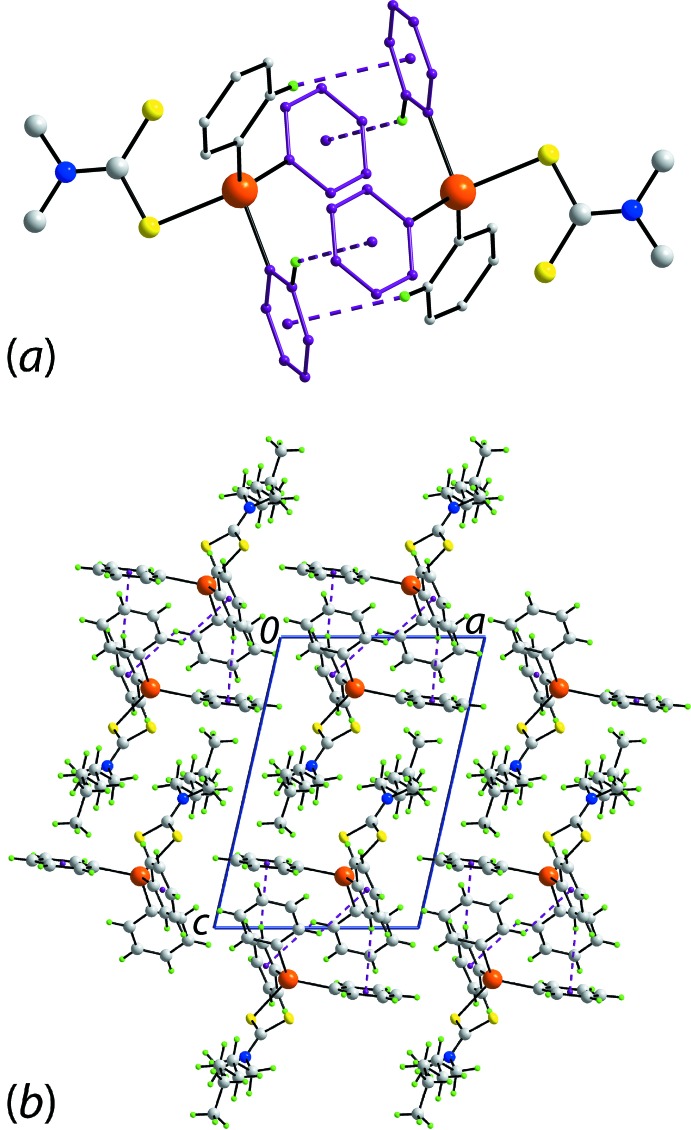
Mol­ecular packing in the crystal of (I): (*a*) supra­molecular dimer sustained by a four-fold embrace of phenyl-C—H⋯π(phen­yl) inter­actions shown as purple dashed lines (for clarity, the phenyl rings are shown as small spheres, the inter­acting phenyl rings are highlighted in purple and only the N-bound carbon atoms of the di­thio­carbamate substituents are shown) and (*b*) a view of the unit-cell contents shown in projection down the *b* axis.

Tables 2 ▸ and 3 ▸ list the geometric parameters characterizing the inter­molecular inter­actions operating in the crystals of (I) and (II), respectively. The mol­ecular packing of (I) features centrosymmetric dimeric aggregates sustained by four phenyl-C—H⋯π(phen­yl) inter­actions whereby all of the participating groups are derived from Sn-bound phenyl rings, Fig. 2 ▸ *a*. Such cooperative C—H⋯π(phen­yl) embraces have been described for many phenyl-rich systems and in instances where six phenyl rings of two residues associate by edge-to-face inter­actions, *i.e*. a six-fold embrace, the energies of stabilization can resemble or even exceed that provided by strong conventional hydrogen bonding (Dance & Scudder, 2009 ▸). The supra­molecular dimers stack parallel to the *b* axis with no directional inter­actions between successive aggregates. Globally, columns pack into layers in the *ab* plane. The layers inter-digitate along the *c* axis, again without specific inter­actions between proximate residues, Fig. 2 ▸ *b*.

**Figure 3:**
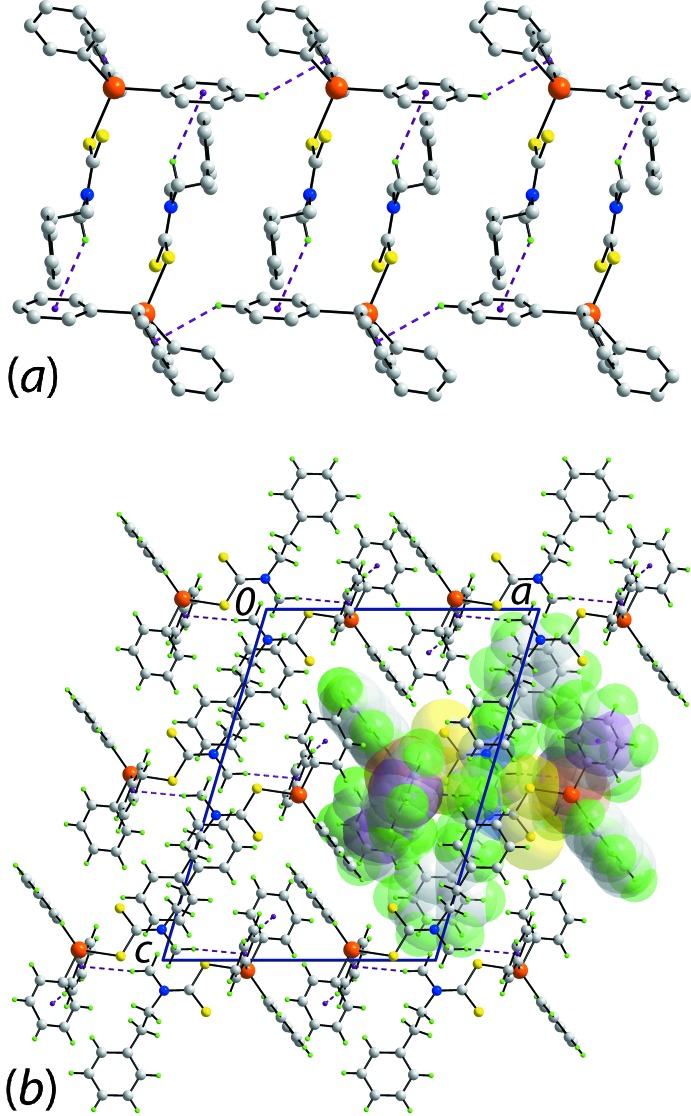
Mol­ecular packing in the crystal of (II): (*a*) supra­molecular chain sustained by C—H⋯π(phen­yl) inter­actions shown as purple dashed lines and (*b*) a view of the unit-cell contents in projection down the *b* axis. One chain is highlighted in space-filling mode.

The mol­ecular packing of (II) again features C—H⋯π inter­actions, as for (I), but with both methyl-H and Sn-bound-H hydrogen atoms as donors; the Sn-phenyl rings function as acceptors. As illustrated in Fig. 3 ▸ *a*, the C—H⋯π inter­actions sustain a supra­molecular chain aligned along the *b* axis. The chains pack into the three-dimensional architecture without directional inter­actions between then, Fig. 3 ▸ *b*. As may be seen from Fig. 3 ▸ *b*, centrosymmetrically related Ph_3_Sn residues approach each other so as to form phenyl-embrace inter­actions as found in the mol­ecular packing of (I), but none of the putative contacts are within the standard distance criteria assumed in *PLATON* (Spek, 2009 ▸).

## Hirshfeld surface analysis   

**Figure 4:**
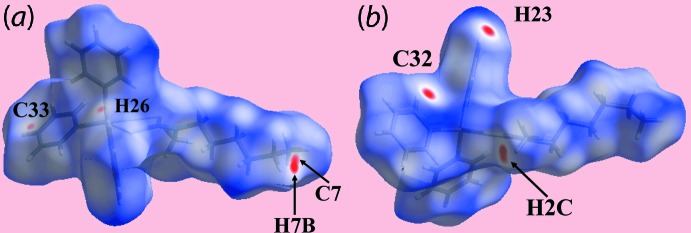
Views of Hirshfeld surface for (I) mapped over *d*_norm_ in the range −0.133 to +1.538 au.

**Figure 5:**
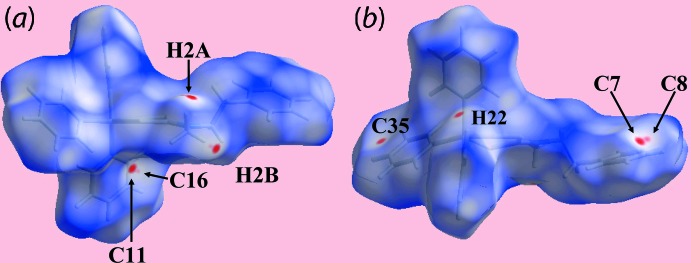
Views of Hirshfeld surface for (II) mapped over *d*_norm_ in the range −0.075 to +1.363 au.

The Hirshfeld surface calculations for the tri­phenyl­tin di­thio­carbamate derivatives (I) and (II) were performed in accord with recent work on related organotin di­thio­carbamates (Mohamad *et al.*, 2017 ▸). Despite the similarity in composition, the structures of (I) and (II) exhibit different inter­molecular environments because of the presence of different substituents in the respective di­thio­carbamate ligands, *i.e. n*-hexyl in the former and phenyl­ethyl in the latter. These differences are readily discerned from the differently shaped Hirshfeld surfaces mapped over *d*_norm_ for (I), Fig. 4 ▸, and (II), Fig. 5 ▸, which reflect the influence of short inter­atomic H⋯H and C⋯H/H⋯C contacts, Table 4 ▸, and comparatively weak C—H⋯π inter­actions, Tables 2 ▸ and 3 ▸.

**Figure 6:**
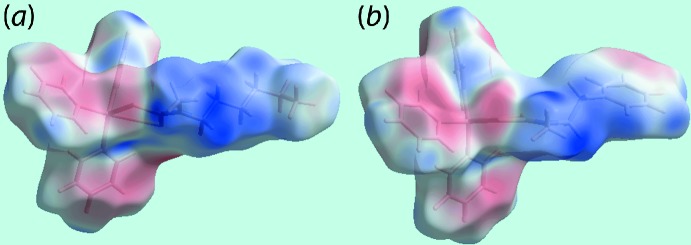
Views of Hirshfeld surface mapped over the electrostatic potential (the red and blue regions represent negative and positive electrostatic potentials, respectively) for a mol­ecule of: (*a*) (I) in the range ±0.041 au and (*b*) (II) in the range −0.033 to +0.049 au.

**Figure 7:**
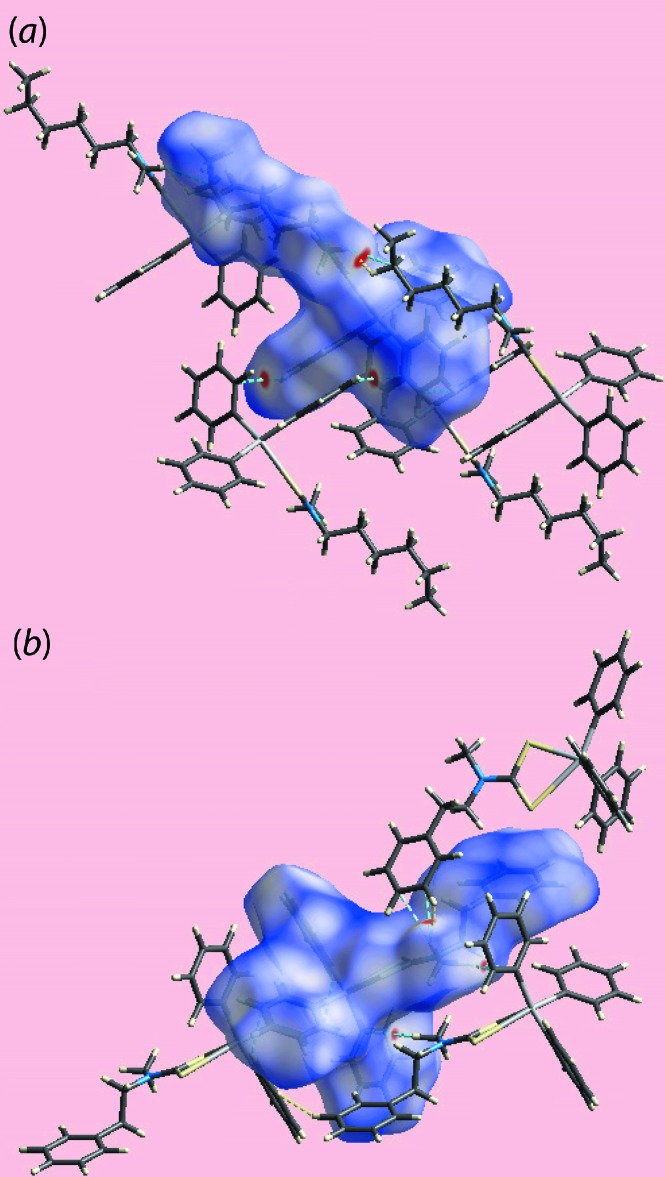
The immediate environment around reference mol­ecules within *d*_norm_-mapped Hirshfeld surfaces for (*a*) (I) and (*b*) (II), highlighting short inter­atomic H⋯H and C⋯H/H⋯C contacts by yellow and blue dashed lines, respectively

The faint-red spots near the phenyl-C33 and H26 atoms in Fig. 4 ▸ *a* reflect the presence of a weak C—H⋯π inter­action, as summarized in Table 4 ▸. In both images of Fig. 4 ▸, the bright-red spots appearing near Sn-bound phenyl atoms C32 and H23, methyl-H2*C* and *n*-hexyl atoms C7 and H7*B* are indicative of the short inter­atomic H⋯H and C⋯H/H⋯C contacts involving these atoms, as listed in Table 4 ▸. The presence of similar inter­molecular inter­actions in the crystal of (II) *cf*. (I), but involving different atoms, is also characterized by bright and faint-red spots on the Hirshfeld surfaces mapped over *d*_norm_ in Fig. 5 ▸. Thus, the C—H⋯π inter­action is seen from the presence of bright-red spots near methyl-H2*B* and phenyl-C11 together with the pair of faint-red spots near the methyl-H2*B* and phenyl-C16 atoms in Fig. 5 ▸ *a*. The influence of other short inter­atomic C⋯H/H⋯C contacts summarized in Table 4 ▸ are viewed as diminutive and faint-red spots near the respective atoms in Fig. 5 ▸ *a*,*b*. The involvement of different atoms in the inter­molecular inter­actions in the crystals of (I) and (II) is also confirmed from the views of their Hirshfeld surfaces mapped over electrostatic potential, Fig. 6 ▸, through the appearance of blue and red regions corresponding to positive and negative electrostatic potentials around them. The different mol­ecular environments about respective reference mol­ecules are highlighted in Fig. 7 ▸.

**Figure 8:**
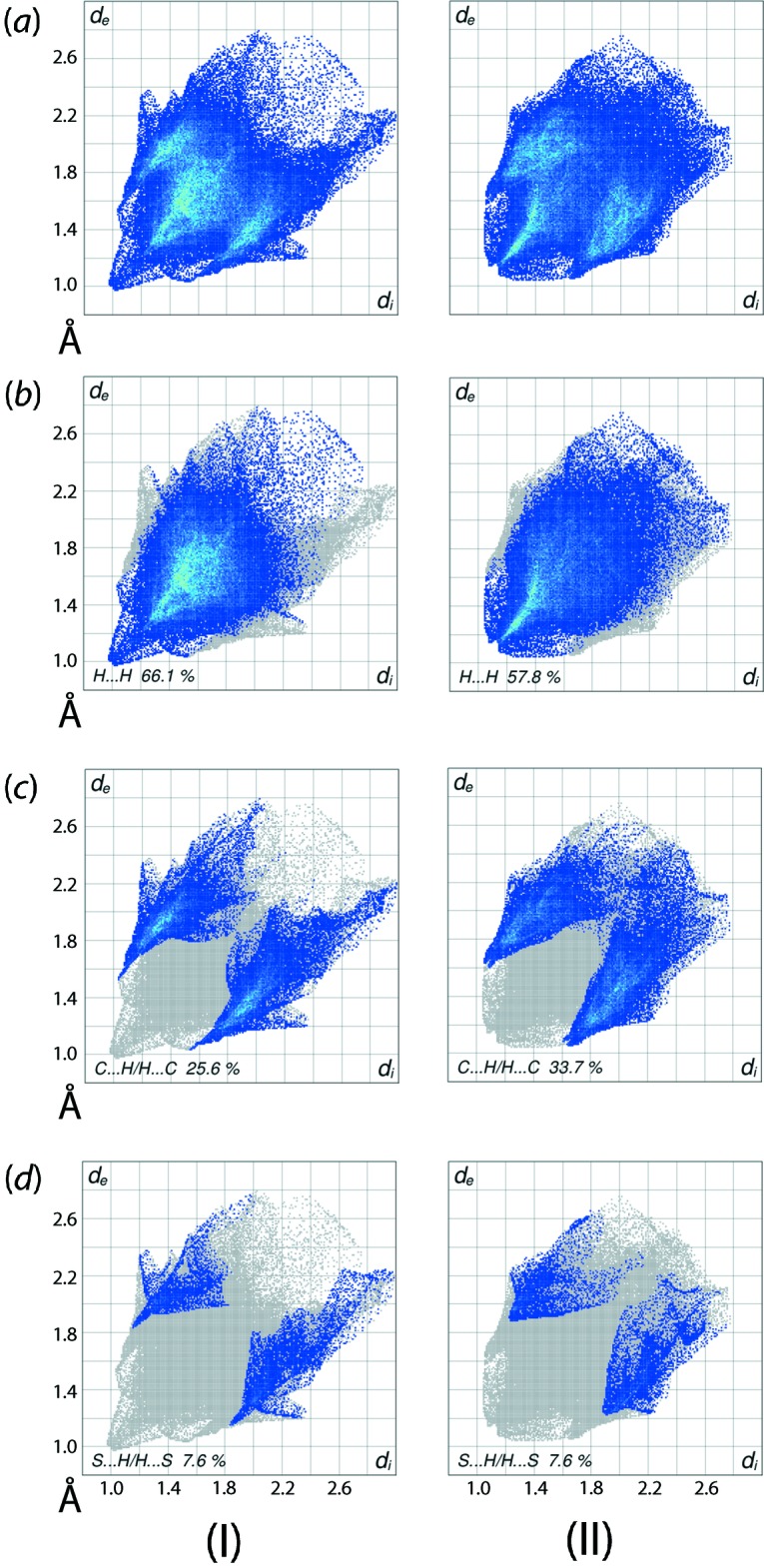
A comparison of the (*a*) full two-dimensional fingerprint plots for (I) and (II), and the plots delineated into (*b*) H⋯H, (*c*) C⋯H/H⋯C and (*d*) S⋯H/H⋯S contacts.

The distinct distribution of points in the overall two-dimensional fingerprint plots for (I) and (II), Fig. 8 ▸ *a*, also highlight the different mol­ecular environments for the two mol­ecules. The significant contributions from H⋯H, C⋯H/H⋯C and S⋯H/H⋯S contacts to the Hirshfeld surfaces of both (I) and (II) are evident from Table 5 ▸. The short inter­atomic H⋯H contact between the methyl-H2*C* and *n*-hexyl-H7*B* atoms in (I) is viewed as a pair of closely spaced overlapping peaks with their tips at *d*_e_ + *d*_i_ ∼2.0 Å in the delineated plot (McKinnon *et al.*, 2007 ▸) Fig. 8 ▸ *b*. A pair of well separated short peaks at *d*_e_ + *d*_i_ ∼2.2 Å observed in the corresponding fingerprint plot for (II) are due to the involvement of methyl-H2*A* and phenyl-H7, H9 and H23 atoms in comparatively weaker short inter­atomic H⋯H contacts, Table 4 ▸. The pair of very thin and long forceps-like tips at *d*_e_ + *d*_i_ ∼2.6 Å in the fingerprint plot delineated into C⋯H/H⋯C contacts for (I), Fig. 8 ▸ *c*, is the result of a short inter­atomic contact between phenyl-C32 and -H23 atoms while the points corresponding to other short inter­atomic contacts are merged within the plot. The presence of a pair of twin forceps-like tips at *d*_e_ + *d*_i_ ∼ 2.7 Å in the C⋯H/H⋯C delineated plot for (II), Fig. 8 ▸ *c*, also indicates the involvement of methyl-H2*A* and -H2*B*, and phenyl-C7, -C8, -C11, -C16 and -C35 atoms in short inter­atomic contacts, Table 4 ▸. Further, it is clear from the fingerprint plots delineated into S⋯H/H⋯S contacts, Fig. 8 ▸ *d*, that the pair of spikes at *d*_e_ + *d*_i_ ∼ 3.0 Å for (I) show van der Waals contacts whereas the pair of peaks at *d*_e_ + *d*_i_ > 3.1 Å for (II) show contacts farther than van der Waals separation. The other inter­atomic contacts summarized in Table 5 ▸ make a negligible contribution to their Hirshfeld surfaces.

## Database survey   

The di­thio­carbamate ligands reported in the present study are quite rare, despite the rather large number of crystal structures of di­thio­carbamate ligands available in the crystallographic literature (Groom *et al.*, 2016 ▸). Thus, the *N*-hexyl-*N*-methyl­dithio­carbamate ligand reported in (I), *i.e*. dtcI, has been reported in the crystal structures of Ph_2_Sn(dtcI)_2_ (Ramasamy *et al.*, 2013 ▸), In(dtcI)_3_ (Park *et al.*, 2003 ▸), and in Bi(dtcI)_3_ and its 1:1 1,10-phenanthroline adduct (Monteiro *et al.*, 2001 ▸). The uniform motivation for these studies was for their evaluation as useful precursors for the deposition of heavy element sulfide nanomaterials. In terms of the mol­ecular structures, no special features in the mode of coordination are noted in the tin (Tiekink, 2008 ▸), indium (Heard, 2005 ▸) and bis­muth (Lai & Tiekink, 2007 ▸) compounds. The *N*-methyl-*N*-phenyl­ethyl­dithio­carbamate ligand, *i.e*. dtcII, has been reported only in its binary mercury(II) compound, *i.e*. Hg(dtcII)_3_ (Green *et al.*, 2004 ▸), and again, its study was motivated by the desire to generate β–HgS thin films and its structure confirms to expectation (Jotani *et al.*, 2016 ▸).

Reflecting the inter­est in organotin di­thio­carbamates, including their biological activity, there are over 50 structures of general formula Ph_3_Sn(S_2_CN*RR*’) in the Cambridge Structural Database (Groom *et al.*, 2016 ▸). Of these, seven are binuclear and are better represented as Ph_3_SnS_2_CN–*R*–NCS_2_SnPh_3_. In all, there are 56 independent coordination geometries and all conform to the same structural motif as described above for (I) and (II). The average Sn—S_short_ bond length is 2.47 Å and the average Sn—S_long_ bond length is 3.04 Å. This gives rise to an average Δ(Sn—S) of 0.57 Å. These values indicate the structures of (I) and (II) are outliers in that the values of Sn—S_long_ are generally longer than usually observed. An analysis of the available crystallographic data showed the shortest Sn—S1 bond length occurred in the structure of Ph_3_Sn(S_2_CNEt_2_) [(III); Hook *et al.* 1994 ▸] while the longest was found for one of the independent tin centres in binuclear Ph_3_Sn[S_2_CN(CH_2_CH_2_)_2_C(H)(CH_2_)_3_C(H)(CH_2_CH_2_)_2_NCS_2_]SnPh_3_ [(IV); Ali *et al.*, 2014 ▸], *i.e*. spanning the range 2.43 to 2.52 Å, Table 6 ▸. The shortest and longest of the Sn⋯S2 separations were found in Ph_3_Sn[S_2_CN(CH_2_Ph)CH_2_CH_2_Ph] [(V); Mohamad, Awang, Kamaludin, Jotani *et al.*, 2016 ▸] and for one of the two independent mol­ecules of Ph_3_Sn{S_2_CN[CH_2_(3-pyrid­yl)]_2_} [(VI); Gupta *et al.*, 2015 ▸], *i.e*. spanning the range 2.91 to 3.22 Å, Table 6 ▸. The lack of systematic variations in these structural parameters is borne out by the disparity of the cited bonds with the second tin centre of non-symmetric (IV) and the second independent mol­ecule of (VI). Thus, the range of Δ(Sn—S) for all structures was 0.40 to 0.74, with the correlation coefficient from the plot of Sn—S_short_ *versus* Sn—S_long_ being 0.52. Such a lack of correlation has often been noted in the structural chemistry of organotin di­thio­carbamates and has been ascribed to the dictates of the mol­ecular packing (Buntine *et al.*, 1998 ▸; Tiekink *et al.*, 1999 ▸; Muthalib *et al.*, 2014 ▸).

## Synthesis and crystallization   

All chemicals and solvents were used as purchased. The melting points were determined using an automated melting-point apparatus (MPA 120 EZ-Melt). C, H, N and S analyses were performed on a Leco CHNS-932 Elemental Analyzer. The IR spectra were obtained on a Perkin Elmer Spectrum GX from 4000 to 400 cm^−1^. NMR spectra were recorded in CDCl_3_ at room temperature on a Bruker AVANCE 400 111 HD.

**Synthesis of tri­phenyl­tin(IV)** ***N*** **-hexyl-** ***N*** **-methyl­dithio­carbamate (I):** *N*-hexyl-*N*-methyl­amine (Aldrich; 1.52 ml, 10 mmol) dissolved in ethanol (30 ml) was stirred at 277 K before a cold ethano­lic solution of carbon di­sulfide (0.6 ml, 10 mmol) was added dropwise. The resulting mixture was stirred for 2 h. Then, tri­phenyl­tin(IV) chloride (Merck; 3.85 g, 10 mmol) dissolved in ethanol (25 ml) was added dropwise into the solution and stirring was continued for 2 h. The precipitate formed was filtered, washed with cold ethanol and dried. Recrystallization was achieved by dissolving the compound in a chloro­form and ethanol mixture (1:1 *v*/*v*). This solution was allowed to slowly evaporate at room temperature yielding colourless slabs of (I). Yield: 52%, m.p. 364.6–365.4 K. Elemental analysis: calculated (%): C 57.8, H 5.8, N 2.6, S 11.9. Found (%): C 56.5, H 6.2, N 2.5, S 11.7. IR (KBr cm^−1^): 1429 ν(C—N), 983 ν(C—S), 559 ν(Sn—C), 425 ν(Sn—S). ^1^H NMR (CDCl_3_): δ 7.41–7.77 (15H, C_6_H_5_); 3.38 (2H, N—CH_2_); 3.42 (3H, N—CH_3_); 2.21 (2H, N—CH_2_C*H*_2_); 1.75 (2H, N—(CH_2_)_2_C*H*_2_); 1.59 (2H, N—(CH_2_)_3_C*H*_2_); 1.34 (2H, N—(CH_2_)_4_C*H*_2_); 0.92 (3H, hex­yl—CH_3_). ^13^C NMR (CDCl_3_): δ 196.04 (NCS_2_); 128.52–142.53 (C-aromatic); 58.97 (NCH_2_); 43.79 (NCH_3_); 31.46 (N—CH_2_ *C*H_2_); 26.98 [N—(CH_2_)_2_ *C*H_2_]; 26.39 [N—(CH_2_)_3_ *C*H_2_]; 22.6 [N—CH_2_)_4_ *C*H_2_]; 14.06 (hex­yl—CH_3_). ^119^Sn NMR (CDCl_3_): −187.56.

**Synthesis of tri­phenyl­tin(IV)** ***N*** **-methyl-** ***N*** **-phenylethyl­dithio­carbamate (II):** compound (II) was prepared in essentially the same manner as for (I) but using *N*-methyl-*N*-phenylethyl­amine (Aldrich; 1.45 ml, 10 mmol) in place of *N*-hexyl-*N*-methyl­amine. Recrystallization was achieved by dissolving the compound in a chloro­form/ethanol mixture (1:2 *v*/*v*). Yield: 67%, m.p. 387.5–388.3 K. Elemental analysis: calculated (%): C 60.0, H 4.9, N 2.5, S 11.4. Found (%): C 57.9, H 5.3, N 2.8, S 11.2. IR (KBr cm^−1^): 1452 ν(C—N), 977 ν(C—S), 502 ν(Sn—C), 488 ν(Sn—S). ^1^H NMR (CDCl_3_): δ 7.43–7.77 (15H, Sn—C_6_H_5_); 7.24–7.35 [5H, N(CH_2_)_2_C_6_ *H*_5_]; 4.06 (2H, NCH_2_); 3.36 (3H, NCH_3_); 3.09 (2H, NCH_2_C*H*_2_). ^13^C NMR (CDCl_3_): δ 196.61 (NCS_2_); 126.8–142.3 (C-aromatic); 60.25 (NCH_2_); 44.59 (NCH_2_ *C*H_2_); 33.12 (N—CH_3_). ^119^Sn NMR (CDCl_3_) = −183.84.

## Refinement   

Crystal data, data collection and structure refinement details are summarized in Table 7 ▸. Carbon-bound H atoms were placed in calculated positions (C—H = 0.95–0.99 Å) and were included in the refinement in the riding-model approximation, with *U*_iso_(H) set to 1.2–1.5*U*_eq_(C). For (I), the maximum and minimum residual electron density peaks of 1.75 and 1.51 e Å^−3^, respectively, are located 0.95 and 0.86 Å from the Sn atom. For (II), the maximum and minimum residual electron density peaks of 1.47 and 1.58 e Å^−3^, respectively, are located 0.96 and 0.68 Å from the C11 and Sn atoms, respectively.

## Supplementary Material

Crystal structure: contains datablock(s) I, II, global. DOI: 10.1107/S2056989018005133/hb7745sup1.cif

Structure factors: contains datablock(s) I. DOI: 10.1107/S2056989018005133/hb7745Isup2.hkl

Structure factors: contains datablock(s) II. DOI: 10.1107/S2056989018005133/hb7745IIsup3.hkl

CCDC references: 1833664, 1833663

Additional supporting information:  crystallographic information; 3D view; checkCIF report

## Figures and Tables

**Table 1:** Selected inter­atomic parameters (Å, °) for (I) and (II)

Parameter	(I)	(II)
Sn—S1	2.4672 (11)	2.4636 (9)
Sn⋯S2	3.1113 (11)	3.1066 (10)
C1—S1	1.761 (4)	1.761 (4)
C1—S2	1.688 (4)	1.678 (4)
C1—N1	1.330 (5)	1.342 (5)
S1—Sn⋯S2	63.26 (3)	63.42 (3)
S1—Sn⋯C11	121.53 (10)	111.30 (9)
S1—Sn⋯C31	90.00 (11)	92.68 (9)
C11—Sn—C21	114.88 (15)	119.27 (13)
S2—Sn⋯C31	152.54 (11)	155.43 (9)

**Table 2:** Hydrogen-bond geometry (Å, °) for (I) *Cg*1 and *Cg*2 are the centroids of the (C11–C16) and (C31–C36) rings, respectively.

*D*—H⋯*A*	*D*—H	H⋯*A*	*D*⋯*A*	*D*—H⋯*A*
C32—H32⋯*Cg*1^i^	0.95	2.88	3.630 (4)	137
C26—H26⋯*Cg*2^i^	0.95	2.99	3.641 (5)	127

Symmetry code: (i) 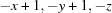.

**Table 3:** Hydrogen-bond geometry (Å, °) for (II) *Cg*1 and *Cg*2 are the ring centroids of the (C11–C16) and (C31–C36) rings, respectively.

*D*—H⋯*A*	*D*—H	H⋯*A*	*D*⋯*A*	*D*—H⋯*A*
C2—H2*B*⋯*Cg*1^i^	0.98	2.99	3.779 (5)	138
C14—H14⋯*Cg*2^ii^	0.95	2.95	3.754 (5)	143

Symmetry codes: (i) 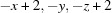; (ii) 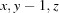.

**Table 4:** Summary of short inter­atomic contacts (Å) in (I) and (II)

Contact	Distance	Symmetry operation
(I)		
H2*C*⋯H7*B*	1.98	*x*, − 1 + *y*, *z*
H2*C*⋯C7	2.67	*x*, − 1 + *y*, *z*
H23⋯C32	2.57	−*x*, − 1 + *y*, − 1 + *z*
H26⋯C33	2.70	1 − *x*, − 1 − *y*, − *z*
		
(II)		
H2*A*⋯H7	2.26	2 − *x*, 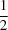 + *y*, 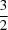 + *z*
H9⋯H23	2.29	1 + *x*, *y*, *z*
H2*A*⋯C7	2.68	2 − *x*, 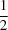 + *y*, 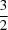 + *z*
H2*A*⋯C8	2.74	2 − *x*, 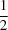 + *y*, 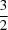 + *z*
H2*B*⋯C11	2.70	2 − *x*, − *y*, 2 − *z*
H2*B*⋯C16	2.77	2 − *x*, − *y*, 2 − *z*
H22⋯C35	2.69	1 − *x*, − *y*, 2 − *z*

**Table 5:** Percentage contributions of inter­atomic contacts to the Hirshfeld surface for (I) and (II)

Contact	Percentage contribution	
	(I)	(II)
H⋯H	66.1	57.8
C⋯H/H⋯C	25.6	33.7
S⋯H/H⋯S	7.6	7.6
N⋯H/H⋯N	0.4	0.6
C⋯N/N⋯C	0.2	0.0
S⋯N/N⋯S	0.1	0.0
C⋯S/S⋯C	0.0	0.3

**Table 6:** Selected inter­atomic parameters (Å) for Ph_3_Sn(S_2_CN*RR*′)

Compound	*R*	*R*′	Sn—S_short_	Sn—S_long_	Δ(Sn—S)	Reference
(III)	Et	Et	2.429 (3)	3.096 (3)	0.67	Hook *et al.* (1994 ▸)
(IV)^*a*^	(CH_2_CH_2_)_2_C(H)CH_2_CH_2_	(CH_2_CH_2_)_2_C(H)CH_2_	2.521 (3)	2.919 (3)	0.40	Ali *et al.* (2014 ▸)
			2.4735 (10)	2.9468 (10)	0.47	
(V)	CH_2_Ph	CH_2_CH_2_Ph	2.4885 (5)	2.9120 (5)	0.42	Mohamad, Awang, Kamaludin, Jotani *et al.* (2016 ▸)
(VI)^*b*^	CH_2_(3-pyrid­yl)	CH_2_(3-pyrid­yl)	2.5165 (19)	3.2209 (19)	0.71	Gupta *et al.* (2015 ▸)
			2.4685 (19)	3.0397 (19)	0.57	

Notes: (*a*) non-symmetric binuclear mol­ecule; (*b*) two independent mol­ecules in the asymmetric unit.

**Table 7:** Experimental details

	(I)	(II)
Crystal data		
Chemical formula	[Sn(C_6_H_5_)_3_(C_8_H_16_NS_2_)]	[Sn(C_6_H_5_)_3_(C_10_H_12_NS_2_)]
*M*_r_	540.38	560.37
Crystal system, space group	Triclinic, *P* 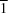	Monoclinic, *P*2_1_/*c*
Temperature (K)	173	173
*a*, *b*, *c* (Å)	9.8590 (6), 10.4256 (5), 14.3960 (8)	14.3682 (4), 9.4758 (2), 19.2747 (6)
α, β, γ (°)	110.557 (5), 94.057 (5), 110.730 (5)	90, 106.450 (3), 90
*V* (Å^3^)	1263.24 (13)	2516.84 (12)
*Z*	2	4
Radiation type	Cu *K*α	Cu *K*α
μ (mm^−1^)	9.67	9.73
Crystal size (mm)	0.30 × 0.20 × 0.05	0.10 × 0.10 × 0.05

Data collection		
Diffractometer	Agilent Technologies SuperNova Dual diffractometer with Atlas detector	Agilent Technologies SuperNova Dual diffractometer with Atlas detector
Absorption correction	Multi-scan (*CrysAlis PRO*; Agilent, 2015 ▸)	Multi-scan (*CrysAlis PRO*; Agilent, 2015 ▸)
*T*_min_, *T*_max_	0.204, 1.000	0.206, 1.000
No. of measured, independent and observed [*I* > 2σ(*I*)] reflections	8833, 5033, 4580	9741, 5054, 4431
*R*_int_	0.057	0.040
(sin θ/λ)_max_ (Å^−1^)	0.628	0.628

Refinement		
*R*[*F* ^2^ > 2σ(*F* ^2^)], *wR*(*F* ^2^), *S*	0.043, 0.121, 1.06	0.039, 0.106, 1.02
No. of reflections	5033	5054
No. of parameters	273	290
H-atom treatment	H-atom parameters constrained	H-atom parameters constrained
Δρ_max_, Δρ_min_ (e Å^−3^)	1.75, −1.51	1.47, −1.58

Computer programs: *CrysAlis PRO* (Agilent, 2015 ▸), *SIR92* (Altomare *et al.*, 1993 ▸), *SHELXL2014* (Sheldrick, 2015 ▸), *ORTEP-3 for Windows* (Farrugia, 2012 ▸), *DIAMOND* (Brandenburg, 2006 ▸) and *publCIF* (Westrip, 2010 ▸).

## supplementary crystallographic information

### (N-Hexyl-N-methyldithiocarbamato-κ2S,S')triphenyltin(IV) (I) Crystal data

**Table:**

[Sn(C_6_H_5_)_3_(C_8_H_16_NS_2_)]	*Z* = 2
*M**_r_* = 540.38	*F*(000) = 552
Triclinic, *P*1	*D*_x_ = 1.421 Mg m^−^^3^
*a* = 9.8590 (6) Å	Cu *K*α radiation, λ = 1.54184 Å
*b* = 10.4256 (5) Å	Cell parameters from 4558 reflections
*c* = 14.3960 (8) Å	θ = 4.7–75.4°
α = 110.557 (5)°	µ = 9.67 mm^−^^1^
β = 94.057 (5)°	*T* = 173 K
γ = 110.730 (5)°	Prism, colourless
*V* = 1263.24 (13) Å^3^	0.30 × 0.20 × 0.05 mm

### (N-Hexyl-N-methyldithiocarbamato-κ2S,S')triphenyltin(IV) (I) Data collection

**Table:**

Agilent Technologies SuperNova Dual diffractometer with Atlas detector	5033 independent reflections
Radiation source: SuperNova (Cu) X-ray Source	4580 reflections with *I* > 2σ(*I*)
Mirror monochromator	*R*_int_ = 0.057
ω scan	θ_max_ = 75.4°, θ_min_ = 4.7°
Absorption correction: multi-scan (CrysAlis PRO; Agilent, 2015)	*h* = −11→12
*T*_min_ = 0.204, *T*_max_ = 1.000	*k* = −12→13
8833 measured reflections	*l* = −17→17

### (N-Hexyl-N-methyldithiocarbamato-κ2S,S')triphenyltin(IV) (I) Refinement

**Table:**

Refinement on *F*^2^	0 restraints
Least-squares matrix: full	Hydrogen site location: inferred from neighbouring sites
*R*[*F*^2^ > 2σ(*F*^2^)] = 0.043	H-atom parameters constrained
*wR*(*F*^2^) = 0.121	*w* = 1/[σ^2^(*F*_o_^2^) + (0.0811*P*)^2^] where *P* = (*F*_o_^2^ + 2*F*_c_^2^)/3
*S* = 1.06	(Δ/σ)_max_ = 0.001
5033 reflections	Δρ_max_ = 1.75 e Å^−^^3^
273 parameters	Δρ_min_ = −1.50 e Å^−^^3^

### (N-Hexyl-N-methyldithiocarbamato-κ2S,S')triphenyltin(IV) (I) Special details

**Table:**

Geometry. All esds (except the esd in the dihedral angle between two l.s. planes) are estimated using the full covariance matrix. The cell esds are taken into account individually in the estimation of esds in distances, angles and torsion angles; correlations between esds in cell parameters are only used when they are defined by crystal symmetry. An approximate (isotropic) treatment of cell esds is used for estimating esds involving l.s. planes.
Refinement. The maximum and minimum residual electron density peaks of 1.75 and 1.50 eÅ^-3^, respectively, were located 0.95 Å and 0.86 Å from the Sn atom.

### (N-Hexyl-N-methyldithiocarbamato-κ2S,S')triphenyltin(IV) (I) Fractional atomic coordinates and isotropic or equivalent isotropic displacement parameters (Å^2^)

**Table:**

	*x*	*y*	*z*	*U*_iso_*/*U*_eq_	
Sn	0.42003 (3)	0.66365 (2)	0.17688 (2)	0.02940 (11)	
S1	0.28273 (12)	0.69634 (11)	0.31417 (8)	0.0359 (2)	
S2	0.46733 (12)	0.99031 (11)	0.31126 (8)	0.0375 (2)	
N1	0.2964 (4)	0.9556 (4)	0.4439 (3)	0.0361 (7)	
C1	0.3470 (4)	0.8930 (4)	0.3645 (3)	0.0311 (8)	
C2	0.1843 (6)	0.8691 (6)	0.4837 (4)	0.0466 (11)	
H2A	0.0966	0.7982	0.4288	0.070*	
H2B	0.1561	0.9368	0.5374	0.070*	
H2C	0.2248	0.8137	0.5119	0.070*	
C3	0.3467 (5)	1.1182 (5)	0.4945 (3)	0.0413 (10)	
H3A	0.4459	1.1674	0.4825	0.050*	
H3B	0.3566	1.1477	0.5688	0.050*	
C4	0.2394 (5)	1.1719 (5)	0.4560 (4)	0.0410 (9)	
H4A	0.1386	1.1175	0.4634	0.049*	
H4B	0.2351	1.1492	0.3828	0.049*	
C5	0.2864 (6)	1.3392 (5)	0.5142 (4)	0.0506 (11)	
H5A	0.3883	1.3936	0.5083	0.061*	
H5B	0.2880	1.3617	0.5871	0.061*	
C6	0.1802 (7)	1.3931 (6)	0.4730 (4)	0.0537 (12)	
H6A	0.1801	1.3713	0.4003	0.064*	
H6B	0.0781	1.3362	0.4774	0.064*	
C7	0.2206 (9)	1.5598 (7)	0.5301 (5)	0.0694 (17)	
H7A	0.3247	1.6157	0.5288	0.083*	
H7B	0.1564	1.5891	0.4932	0.083*	
C8	0.2058 (9)	1.6078 (7)	0.6395 (5)	0.0719 (18)	
H8A	0.1058	1.5468	0.6426	0.108*	
H8B	0.2218	1.7137	0.6672	0.108*	
H8C	0.2800	1.5941	0.6795	0.108*	
C11	0.6597 (4)	0.7469 (4)	0.2096 (3)	0.0304 (7)	
C12	0.7510 (5)	0.8927 (5)	0.2260 (3)	0.0379 (9)	
H12	0.7089	0.9628	0.2292	0.045*	
C13	0.9038 (5)	0.9364 (5)	0.2376 (4)	0.0447 (10)	
H13	0.9657	1.0367	0.2495	0.054*	
C14	0.9665 (5)	0.8351 (6)	0.2318 (4)	0.0454 (10)	
H14	1.0710	0.8654	0.2394	0.054*	
C15	0.8759 (5)	0.6892 (6)	0.2148 (4)	0.0427 (10)	
H15	0.9188	0.6195	0.2113	0.051*	
C16	0.7237 (5)	0.6441 (5)	0.2028 (3)	0.0356 (8)	
H16	0.6622	0.5433	0.1900	0.043*	
C21	0.3260 (4)	0.7039 (4)	0.0583 (3)	0.0286 (7)	
C22	0.1745 (5)	0.6661 (5)	0.0340 (4)	0.0404 (9)	
H22	0.1116	0.6201	0.0704	0.048*	
C23	0.1134 (6)	0.6942 (6)	−0.0422 (4)	0.0513 (12)	
H23	0.0091	0.6666	−0.0583	0.062*	
C24	0.2042 (6)	0.7626 (6)	−0.0950 (4)	0.0486 (11)	
H24	0.1623	0.7819	−0.1474	0.058*	
C25	0.3532 (6)	0.8021 (6)	−0.0721 (4)	0.0491 (11)	
H25	0.4154	0.8502	−0.1079	0.059*	
C26	0.4154 (5)	0.7723 (5)	0.0041 (3)	0.0396 (9)	
H26	0.5196	0.7990	0.0190	0.047*	
C31	0.3442 (4)	0.4284 (4)	0.1449 (3)	0.0300 (7)	
C32	0.2560 (5)	0.3130 (5)	0.0524 (3)	0.0359 (8)	
H32	0.2296	0.3366	−0.0024	0.043*	
C33	0.2063 (5)	0.1638 (5)	0.0394 (4)	0.0468 (11)	
H33	0.1433	0.0870	−0.0233	0.056*	
C34	0.2475 (6)	0.1261 (5)	0.1167 (4)	0.0519 (12)	
H34	0.2146	0.0240	0.1071	0.062*	
C35	0.3367 (6)	0.2384 (6)	0.2077 (4)	0.0503 (12)	
H35	0.3673	0.2135	0.2607	0.060*	
C36	0.3824 (5)	0.3874 (5)	0.2229 (4)	0.0423 (10)	
H36	0.4408	0.4634	0.2872	0.051*	

### (N-Hexyl-N-methyldithiocarbamato-κ2S,S')triphenyltin(IV) (I) Atomic displacement parameters (Å^2^)

**Table:**

	*U*^11^	*U*^22^	*U*^33^	*U*^12^	*U*^13^	*U*^23^
Sn	0.02606 (15)	0.03203 (15)	0.03534 (16)	0.01310 (11)	0.00868 (10)	0.01771 (11)
S1	0.0372 (5)	0.0344 (4)	0.0426 (5)	0.0156 (4)	0.0165 (4)	0.0201 (4)
S2	0.0369 (5)	0.0362 (5)	0.0465 (5)	0.0170 (4)	0.0175 (4)	0.0208 (4)
N1	0.0326 (18)	0.0422 (18)	0.0386 (17)	0.0188 (15)	0.0119 (14)	0.0177 (15)
C1	0.0272 (18)	0.0334 (18)	0.0350 (18)	0.0149 (15)	0.0058 (15)	0.0139 (15)
C2	0.056 (3)	0.049 (2)	0.049 (2)	0.027 (2)	0.030 (2)	0.027 (2)
C3	0.041 (2)	0.042 (2)	0.039 (2)	0.0205 (19)	0.0047 (18)	0.0101 (18)
C4	0.038 (2)	0.040 (2)	0.049 (2)	0.0189 (18)	0.0124 (19)	0.0191 (19)
C5	0.053 (3)	0.040 (2)	0.060 (3)	0.020 (2)	0.017 (2)	0.019 (2)
C6	0.070 (3)	0.052 (3)	0.053 (3)	0.032 (3)	0.022 (2)	0.028 (2)
C7	0.095 (5)	0.055 (3)	0.084 (4)	0.041 (3)	0.044 (4)	0.042 (3)
C8	0.109 (6)	0.062 (3)	0.066 (4)	0.049 (4)	0.028 (4)	0.033 (3)
C11	0.0265 (18)	0.0358 (18)	0.0315 (17)	0.0131 (15)	0.0086 (14)	0.0155 (15)
C12	0.033 (2)	0.038 (2)	0.044 (2)	0.0159 (17)	0.0089 (17)	0.0158 (17)
C13	0.031 (2)	0.045 (2)	0.052 (2)	0.0098 (18)	0.0079 (19)	0.018 (2)
C14	0.026 (2)	0.056 (3)	0.049 (2)	0.0161 (19)	0.0061 (18)	0.017 (2)
C15	0.036 (2)	0.056 (3)	0.044 (2)	0.028 (2)	0.0103 (18)	0.020 (2)
C16	0.036 (2)	0.042 (2)	0.0357 (19)	0.0205 (17)	0.0106 (16)	0.0192 (17)
C21	0.0264 (18)	0.0300 (16)	0.0332 (17)	0.0134 (14)	0.0054 (14)	0.0154 (14)
C22	0.0256 (19)	0.052 (2)	0.050 (2)	0.0162 (17)	0.0109 (17)	0.026 (2)
C23	0.034 (2)	0.070 (3)	0.056 (3)	0.026 (2)	0.004 (2)	0.027 (2)
C24	0.056 (3)	0.058 (3)	0.044 (2)	0.033 (2)	0.005 (2)	0.024 (2)
C25	0.058 (3)	0.060 (3)	0.052 (3)	0.031 (2)	0.023 (2)	0.039 (2)
C26	0.030 (2)	0.052 (2)	0.047 (2)	0.0187 (18)	0.0131 (17)	0.027 (2)
C31	0.0247 (17)	0.0306 (17)	0.0366 (18)	0.0113 (14)	0.0093 (15)	0.0151 (15)
C32	0.030 (2)	0.041 (2)	0.042 (2)	0.0147 (17)	0.0176 (16)	0.0204 (17)
C33	0.042 (2)	0.035 (2)	0.049 (2)	0.0056 (18)	0.018 (2)	0.0105 (19)
C34	0.056 (3)	0.033 (2)	0.073 (3)	0.017 (2)	0.033 (3)	0.027 (2)
C35	0.059 (3)	0.052 (3)	0.063 (3)	0.030 (2)	0.026 (2)	0.039 (2)
C36	0.049 (3)	0.037 (2)	0.048 (2)	0.0188 (19)	0.010 (2)	0.0238 (18)

### (N-Hexyl-N-methyldithiocarbamato-κ2S,S')triphenyltin(IV) (I) Geometric parameters (Å, º)

**Table:**

Sn—C21	2.123 (4)	C11—C16	1.404 (6)
Sn—C31	2.156 (4)	C12—C13	1.389 (6)
Sn—C11	2.159 (4)	C12—H12	0.9500
Sn—S1	2.4672 (11)	C13—C14	1.383 (7)
Sn—S2	3.1113 (11)	C13—H13	0.9500
S1—C1	1.761 (4)	C14—C15	1.384 (7)
S2—C1	1.688 (4)	C14—H14	0.9500
N1—C1	1.330 (5)	C15—C16	1.382 (6)
N1—C2	1.453 (6)	C15—H15	0.9500
N1—C3	1.462 (6)	C16—H16	0.9500
C2—H2A	0.9800	C21—C22	1.386 (5)
C2—H2B	0.9800	C21—C26	1.392 (6)
C2—H2C	0.9800	C22—C23	1.382 (7)
C3—C4	1.514 (6)	C22—H22	0.9500
C3—H3A	0.9900	C23—C24	1.383 (8)
C3—H3B	0.9900	C23—H23	0.9500
C4—C5	1.518 (6)	C24—C25	1.358 (7)
C4—H4A	0.9900	C24—H24	0.9500
C4—H4B	0.9900	C25—C26	1.398 (6)
C5—C6	1.526 (7)	C25—H25	0.9500
C5—H5A	0.9900	C26—H26	0.9500
C5—H5B	0.9900	C31—C32	1.396 (6)
C6—C7	1.521 (8)	C31—C36	1.405 (6)
C6—H6A	0.9900	C32—C33	1.392 (6)
C6—H6B	0.9900	C32—H32	0.9500
C7—C8	1.510 (9)	C33—C34	1.385 (8)
C7—H7A	0.9900	C33—H33	0.9500
C7—H7B	0.9900	C34—C35	1.375 (8)
C8—H8A	0.9800	C34—H34	0.9500
C8—H8B	0.9800	C35—C36	1.385 (6)
C8—H8C	0.9800	C35—H35	0.9500
C11—C12	1.388 (6)	C36—H36	0.9500
			
C21—Sn—C31	112.73 (14)	C7—C8—H8C	109.5
C21—Sn—C11	114.88 (15)	H8A—C8—H8C	109.5
C31—Sn—C11	104.73 (15)	H8B—C8—H8C	109.5
C21—Sn—S1	109.94 (11)	C12—C11—C16	119.1 (4)
C31—Sn—S1	90.00 (11)	C12—C11—Sn	122.6 (3)
C11—Sn—S1	121.53 (10)	C16—C11—Sn	117.9 (3)
C21—Sn—S2	84.08 (10)	C11—C12—C13	120.2 (4)
C31—Sn—S2	152.54 (11)	C11—C12—H12	119.9
C11—Sn—S2	85.86 (11)	C13—C12—H12	119.9
S1—Sn—S2	63.26 (3)	C14—C13—C12	120.5 (4)
C1—S1—Sn	98.62 (14)	C14—C13—H13	119.7
C1—S2—Sn	78.80 (14)	C12—C13—H13	119.7
C1—N1—C2	123.0 (4)	C13—C14—C15	119.5 (4)
C1—N1—C3	121.5 (4)	C13—C14—H14	120.2
C2—N1—C3	115.4 (4)	C15—C14—H14	120.2
N1—C1—S2	124.0 (3)	C16—C15—C14	120.5 (4)
N1—C1—S1	116.7 (3)	C16—C15—H15	119.7
S2—C1—S1	119.3 (2)	C14—C15—H15	119.7
N1—C2—H2A	109.5	C15—C16—C11	120.1 (4)
N1—C2—H2B	109.5	C15—C16—H16	119.9
H2A—C2—H2B	109.5	C11—C16—H16	119.9
N1—C2—H2C	109.5	C22—C21—C26	118.0 (4)
H2A—C2—H2C	109.5	C22—C21—Sn	121.1 (3)
H2B—C2—H2C	109.5	C26—C21—Sn	120.9 (3)
N1—C3—C4	111.7 (4)	C23—C22—C21	121.2 (4)
N1—C3—H3A	109.3	C23—C22—H22	119.4
C4—C3—H3A	109.3	C21—C22—H22	119.4
N1—C3—H3B	109.3	C24—C23—C22	119.9 (4)
C4—C3—H3B	109.3	C24—C23—H23	120.1
H3A—C3—H3B	107.9	C22—C23—H23	120.1
C3—C4—C5	111.4 (4)	C25—C24—C23	120.1 (4)
C3—C4—H4A	109.3	C25—C24—H24	119.9
C5—C4—H4A	109.4	C23—C24—H24	119.9
C3—C4—H4B	109.3	C24—C25—C26	120.2 (5)
C5—C4—H4B	109.4	C24—C25—H25	119.9
H4A—C4—H4B	108.0	C26—C25—H25	119.9
C4—C5—C6	111.1 (4)	C21—C26—C25	120.5 (4)
C4—C5—H5A	109.4	C21—C26—H26	119.7
C6—C5—H5A	109.4	C25—C26—H26	119.7
C4—C5—H5B	109.4	C32—C31—C36	117.4 (4)
C6—C5—H5B	109.4	C32—C31—Sn	124.4 (3)
H5A—C5—H5B	108.0	C36—C31—Sn	118.2 (3)
C7—C6—C5	113.2 (5)	C33—C32—C31	120.8 (4)
C7—C6—H6A	108.9	C33—C32—H32	119.6
C5—C6—H6A	108.9	C31—C32—H32	119.6
C7—C6—H6B	108.9	C34—C33—C32	120.8 (4)
C5—C6—H6B	108.9	C34—C33—H33	119.6
H6A—C6—H6B	107.8	C32—C33—H33	119.6
C8—C7—C6	115.3 (5)	C35—C34—C33	119.1 (4)
C8—C7—H7A	108.4	C35—C34—H34	120.5
C6—C7—H7A	108.4	C33—C34—H34	120.5
C8—C7—H7B	108.4	C34—C35—C36	120.7 (5)
C6—C7—H7B	108.4	C34—C35—H35	119.7
H7A—C7—H7B	107.5	C36—C35—H35	119.7
C7—C8—H8A	109.5	C35—C36—C31	121.2 (4)
C7—C8—H8B	109.5	C35—C36—H36	119.4
H8A—C8—H8B	109.5	C31—C36—H36	119.4
			
C2—N1—C1—S2	175.2 (3)	C14—C15—C16—C11	−1.1 (7)
C3—N1—C1—S2	−2.3 (6)	C12—C11—C16—C15	1.5 (6)
C2—N1—C1—S1	−5.0 (5)	Sn—C11—C16—C15	174.7 (3)
C3—N1—C1—S1	177.6 (3)	C26—C21—C22—C23	0.5 (7)
Sn—S2—C1—N1	179.5 (4)	Sn—C21—C22—C23	179.0 (4)
Sn—S2—C1—S1	−0.32 (19)	C21—C22—C23—C24	−0.7 (8)
Sn—S1—C1—N1	−179.4 (3)	C22—C23—C24—C25	0.0 (8)
Sn—S1—C1—S2	0.4 (2)	C23—C24—C25—C26	0.8 (8)
C1—N1—C3—C4	96.6 (5)	C22—C21—C26—C25	0.2 (7)
C2—N1—C3—C4	−81.0 (5)	Sn—C21—C26—C25	−178.3 (4)
N1—C3—C4—C5	175.9 (4)	C24—C25—C26—C21	−0.9 (8)
C3—C4—C5—C6	178.5 (4)	C36—C31—C32—C33	1.2 (6)
C4—C5—C6—C7	178.9 (5)	Sn—C31—C32—C33	−177.2 (3)
C5—C6—C7—C8	−66.4 (8)	C31—C32—C33—C34	−2.3 (7)
C16—C11—C12—C13	−1.3 (6)	C32—C33—C34—C35	1.0 (8)
Sn—C11—C12—C13	−174.2 (3)	C33—C34—C35—C36	1.3 (8)
C11—C12—C13—C14	0.7 (7)	C34—C35—C36—C31	−2.5 (8)
C12—C13—C14—C15	−0.3 (7)	C32—C31—C36—C35	1.2 (7)
C13—C14—C15—C16	0.5 (7)	Sn—C31—C36—C35	179.6 (4)

### (N-Hexyl-N-methyldithiocarbamato-κ2S,S')triphenyltin(IV) (I) Hydrogen-bond geometry (Å, º)

Cg1 and Cg2 are the centroids of the (C11–C16) and (C31–C36) rings, respectively.

**Table:**

*D*—H···*A*	*D*—H	H···*A*	*D*···*A*	*D*—H···*A*
C32—H32···*Cg*1^i^	0.95	2.88	3.630 (4)	137
C26—H26···*Cg*2^i^	0.95	2.99	3.641 (5)	127

Symmetry code: (i) −*x*+1, −*y*+1, −*z*.

### [N-Methyl-N-(2-phenylethyl)dithiocarbamato-κ2S,S']triphenyltin(IV) (II) Crystal data

**Table:**

[Sn(C_6_H_5_)_3_(C_10_H_12_NS_2_)]	*F*(000) = 1136
*M**_r_* = 560.37	*D*_x_ = 1.479 Mg m^−^^3^
Monoclinic, *P*2_1_/*c*	Cu *K*α radiation, λ = 1.54184 Å
*a* = 14.3682 (4) Å	Cell parameters from 4369 reflections
*b* = 9.4758 (2) Å	θ = 4.8–75.1°
*c* = 19.2747 (6) Å	µ = 9.73 mm^−^^1^
β = 106.450 (3)°	*T* = 173 K
*V* = 2516.84 (12) Å^3^	Prism, colourless
*Z* = 4	0.10 × 0.10 × 0.05 mm

### [N-Methyl-N-(2-phenylethyl)dithiocarbamato-κ2S,S']triphenyltin(IV) (II) Data collection

**Table:**

Agilent Technologies SuperNova Dual diffractometer with Atlas detector	5054 independent reflections
Radiation source: SuperNova (Cu) X-ray Source	4431 reflections with *I* > 2σ(*I*)
Mirror monochromator	*R*_int_ = 0.040
Detector resolution: 10.4041 pixels mm^-1^	θ_max_ = 75.6°, θ_min_ = 4.8°
ω scan	*h* = −17→13
Absorption correction: multi-scan (CrysAlis PRO; Agilent, 2015)	*k* = −11→7
*T*_min_ = 0.206, *T*_max_ = 1.000	*l* = −22→24
9741 measured reflections	

### [N-Methyl-N-(2-phenylethyl)dithiocarbamato-κ2S,S']triphenyltin(IV) (II) Refinement

**Table:**

Refinement on *F*^2^	0 restraints
Least-squares matrix: full	Hydrogen site location: inferred from neighbouring sites
*R*[*F*^2^ > 2σ(*F*^2^)] = 0.039	H-atom parameters constrained
*wR*(*F*^2^) = 0.106	*w* = 1/[σ^2^(*F*_o_^2^) + (0.0648*P*)^2^] where *P* = (*F*_o_^2^ + 2*F*_c_^2^)/3
*S* = 1.02	(Δ/σ)_max_ = 0.003
5054 reflections	Δρ_max_ = 1.47 e Å^−^^3^
290 parameters	Δρ_min_ = −1.58 e Å^−^^3^

### [N-Methyl-N-(2-phenylethyl)dithiocarbamato-κ2S,S']triphenyltin(IV) (II) Special details

**Table:**

Geometry. All esds (except the esd in the dihedral angle between two l.s. planes) are estimated using the full covariance matrix. The cell esds are taken into account individually in the estimation of esds in distances, angles and torsion angles; correlations between esds in cell parameters are only used when they are defined by crystal symmetry. An approximate (isotropic) treatment of cell esds is used for estimating esds involving l.s. planes.
Refinement. The maximum and minimum residual electron density peaks of 1.47 and 1.58 eÅ^-3^, respectively, were located 0.96 Å and 0.68 Å from the C11 and Sn atoms, respectively.

### [N-Methyl-N-(2-phenylethyl)dithiocarbamato-κ2S,S']triphenyltin(IV) (II) Fractional atomic coordinates and isotropic or equivalent isotropic displacement parameters (Å^2^)

**Table:**

	*x*	*y*	*z*	*U*_iso_*/*U*_eq_	
Sn	0.68172 (2)	0.06519 (2)	0.97084 (2)	0.02404 (9)	
S1	0.84111 (6)	0.17866 (9)	0.98902 (5)	0.0307 (2)	
S2	0.78587 (7)	0.01259 (10)	0.85224 (5)	0.0350 (2)	
N1	0.9625 (2)	0.1189 (3)	0.91290 (17)	0.0320 (7)	
C1	0.8706 (3)	0.1012 (4)	0.91495 (19)	0.0279 (7)	
C2	1.0356 (3)	0.1925 (4)	0.9697 (2)	0.0369 (8)	
H2A	1.0283	0.2946	0.9619	0.055*	
H2B	1.1005	0.1636	0.9683	0.055*	
H2C	1.0270	0.1687	1.0170	0.055*	
C3	0.9948 (3)	0.0678 (4)	0.8520 (2)	0.0344 (9)	
H3A	0.9483	−0.0036	0.8248	0.041*	
H3B	1.0589	0.0216	0.8705	0.041*	
C4	1.0026 (3)	0.1877 (4)	0.8013 (2)	0.0427 (10)	
H4A	0.9398	0.2383	0.7856	0.051*	
H4B	1.0527	0.2555	0.8275	0.051*	
C5	1.0287 (3)	0.1338 (4)	0.7357 (2)	0.0353 (8)	
C6	0.9574 (3)	0.0996 (4)	0.6730 (2)	0.0386 (9)	
H6	0.8911	0.1128	0.6709	0.046*	
C7	0.9812 (3)	0.0467 (4)	0.6137 (2)	0.0400 (9)	
H7	0.9311	0.0239	0.5712	0.048*	
C8	1.0764 (3)	0.0264 (5)	0.6150 (2)	0.0401 (9)	
H8	1.0924	−0.0106	0.5740	0.048*	
C9	1.1483 (3)	0.0605 (6)	0.6769 (3)	0.0513 (13)	
H9	1.2144	0.0472	0.6784	0.062*	
C10	1.1254 (3)	0.1139 (6)	0.7368 (2)	0.0469 (11)	
H10	1.1759	0.1372	0.7790	0.056*	
C11	0.6968 (2)	−0.1552 (3)	0.9966 (2)	0.0245 (7)	
C12	0.6890 (3)	−0.1965 (4)	1.0642 (2)	0.0311 (8)	
H12	0.6760	−0.1278	1.0961	0.037*	
C13	0.7002 (3)	−0.3379 (4)	1.0855 (2)	0.0392 (9)	
H13	0.6963	−0.3644	1.1321	0.047*	
C14	0.7168 (3)	−0.4394 (4)	1.0389 (3)	0.0377 (9)	
H14	0.7242	−0.5356	1.0533	0.045*	
C15	0.7227 (3)	−0.4001 (4)	0.9714 (2)	0.0344 (8)	
H15	0.7331	−0.4699	0.9390	0.041*	
C16	0.7137 (3)	−0.2591 (4)	0.9504 (2)	0.0308 (8)	
H16	0.7190	−0.2334	0.9040	0.037*	
C21	0.5674 (2)	0.1332 (3)	0.88077 (18)	0.0238 (7)	
C22	0.5054 (3)	0.0368 (4)	0.8369 (2)	0.0351 (8)	
H22	0.5164	−0.0613	0.8457	0.042*	
C23	0.4274 (3)	0.0811 (5)	0.7804 (2)	0.0424 (10)	
H23	0.3857	0.0136	0.7507	0.051*	
C24	0.4107 (3)	0.2222 (5)	0.7676 (2)	0.0414 (10)	
H24	0.3568	0.2528	0.7294	0.050*	
C25	0.4718 (4)	0.3196 (4)	0.8099 (2)	0.0449 (10)	
H25	0.4606	0.4174	0.8003	0.054*	
C26	0.5496 (3)	0.2767 (4)	0.8665 (2)	0.0366 (9)	
H26	0.5912	0.3450	0.8957	0.044*	
C31	0.6570 (2)	0.1695 (4)	1.06344 (19)	0.0262 (7)	
C32	0.7271 (3)	0.1666 (4)	1.1310 (2)	0.0311 (8)	
H32	0.7863	0.1174	1.1362	0.037*	
C33	0.7110 (3)	0.2349 (4)	1.1904 (2)	0.0331 (8)	
H33	0.7588	0.2309	1.2360	0.040*	
C34	0.6260 (3)	0.3084 (4)	1.1836 (2)	0.0328 (8)	
H34	0.6152	0.3545	1.2244	0.039*	
C35	0.5566 (3)	0.3150 (4)	1.1174 (2)	0.0348 (8)	
H35	0.4986	0.3670	1.1125	0.042*	
C36	0.5718 (3)	0.2455 (4)	1.0579 (2)	0.0284 (7)	
H36	0.5232	0.2497	1.0127	0.034*	

### [N-Methyl-N-(2-phenylethyl)dithiocarbamato-κ2S,S']triphenyltin(IV) (II) Atomic displacement parameters (Å^2^)

**Table:**

	*U*^11^	*U*^22^	*U*^33^	*U*^12^	*U*^13^	*U*^23^
Sn	0.02450 (14)	0.02123 (14)	0.02577 (14)	0.00113 (8)	0.00611 (10)	−0.00044 (8)
S1	0.0308 (4)	0.0320 (4)	0.0324 (4)	−0.0046 (3)	0.0139 (4)	−0.0054 (3)
S2	0.0352 (5)	0.0397 (5)	0.0307 (5)	−0.0032 (4)	0.0104 (4)	−0.0035 (4)
N1	0.0303 (16)	0.0367 (17)	0.0309 (16)	0.0030 (13)	0.0117 (14)	0.0010 (13)
C1	0.0307 (19)	0.0294 (17)	0.0279 (17)	0.0023 (15)	0.0152 (15)	0.0038 (14)
C2	0.0300 (19)	0.044 (2)	0.036 (2)	0.0008 (17)	0.0076 (17)	0.0023 (17)
C3	0.031 (2)	0.037 (2)	0.042 (2)	0.0049 (15)	0.0213 (18)	0.0011 (16)
C4	0.056 (3)	0.038 (2)	0.039 (2)	0.0026 (19)	0.022 (2)	0.0010 (17)
C5	0.043 (2)	0.0305 (19)	0.035 (2)	0.0003 (16)	0.0158 (18)	0.0041 (15)
C6	0.033 (2)	0.039 (2)	0.044 (2)	0.0091 (17)	0.0105 (19)	0.0015 (18)
C7	0.037 (2)	0.041 (2)	0.038 (2)	0.0040 (17)	0.0031 (19)	−0.0035 (17)
C8	0.041 (2)	0.049 (2)	0.033 (2)	−0.0006 (19)	0.0169 (18)	0.0011 (18)
C9	0.029 (2)	0.086 (4)	0.041 (3)	−0.006 (2)	0.015 (2)	0.001 (2)
C10	0.036 (2)	0.073 (3)	0.031 (2)	−0.011 (2)	0.0091 (18)	−0.003 (2)
C11	0.0182 (15)	0.0191 (15)	0.0334 (18)	0.0001 (12)	0.0029 (14)	0.0021 (13)
C12	0.0356 (19)	0.0228 (16)	0.038 (2)	0.0012 (14)	0.0153 (17)	0.0001 (14)
C13	0.045 (2)	0.034 (2)	0.042 (2)	0.0025 (18)	0.0184 (19)	0.0113 (17)
C14	0.037 (2)	0.0227 (18)	0.057 (3)	0.0018 (14)	0.020 (2)	0.0058 (16)
C15	0.031 (2)	0.0254 (17)	0.048 (2)	−0.0052 (15)	0.0145 (18)	−0.0101 (16)
C16	0.0303 (18)	0.0284 (17)	0.0335 (19)	−0.0042 (15)	0.0087 (16)	−0.0026 (15)
C21	0.0246 (16)	0.0227 (16)	0.0241 (16)	0.0022 (13)	0.0067 (14)	0.0012 (12)
C22	0.040 (2)	0.0238 (17)	0.040 (2)	−0.0031 (16)	0.0078 (18)	0.0025 (15)
C23	0.033 (2)	0.051 (3)	0.035 (2)	−0.0049 (18)	−0.0032 (18)	−0.0001 (18)
C24	0.038 (2)	0.055 (3)	0.030 (2)	0.0156 (19)	0.0076 (18)	0.0075 (18)
C25	0.060 (3)	0.030 (2)	0.042 (2)	0.0186 (19)	0.010 (2)	0.0098 (17)
C26	0.048 (2)	0.0220 (17)	0.034 (2)	0.0027 (16)	0.0033 (18)	0.0005 (14)
C31	0.0257 (17)	0.0235 (16)	0.0295 (17)	−0.0039 (14)	0.0080 (15)	−0.0034 (13)
C32	0.0314 (18)	0.0293 (18)	0.0326 (19)	0.0040 (15)	0.0092 (16)	−0.0026 (15)
C33	0.0330 (19)	0.0358 (19)	0.0277 (18)	−0.0020 (16)	0.0043 (16)	−0.0078 (15)
C34	0.037 (2)	0.0248 (17)	0.041 (2)	−0.0015 (15)	0.0173 (17)	−0.0106 (15)
C35	0.0273 (18)	0.0295 (18)	0.050 (2)	0.0024 (15)	0.0149 (17)	−0.0056 (16)
C36	0.0216 (16)	0.0292 (17)	0.0318 (18)	−0.0008 (14)	0.0032 (14)	−0.0025 (14)

### [N-Methyl-N-(2-phenylethyl)dithiocarbamato-κ2S,S']triphenyltin(IV) (II) Geometric parameters (Å, º)

**Table:**

Sn—C21	2.125 (3)	C12—C13	1.397 (5)
Sn—C11	2.143 (3)	C12—H12	0.9500
Sn—C31	2.156 (3)	C13—C14	1.382 (6)
Sn—S1	2.4636 (9)	C13—H13	0.9500
Sn—S2	3.1066 (10)	C14—C15	1.379 (6)
S1—C1	1.761 (4)	C14—H14	0.9500
S2—C1	1.678 (4)	C15—C16	1.392 (5)
N1—C1	1.342 (5)	C15—H15	0.9500
N1—C2	1.462 (5)	C16—H16	0.9500
N1—C3	1.462 (5)	C21—C22	1.384 (5)
C2—H2A	0.9800	C21—C26	1.396 (5)
C2—H2B	0.9800	C22—C23	1.389 (6)
C2—H2C	0.9800	C22—H22	0.9500
C3—C4	1.524 (5)	C23—C24	1.369 (6)
C3—H3A	0.9900	C23—H23	0.9500
C3—H3B	0.9900	C24—C25	1.373 (6)
C4—C5	1.505 (5)	C24—H24	0.9500
C4—H4A	0.9900	C25—C26	1.383 (6)
C4—H4B	0.9900	C25—H25	0.9500
C5—C6	1.384 (6)	C26—H26	0.9500
C5—C10	1.396 (6)	C31—C32	1.403 (5)
C6—C7	1.378 (6)	C31—C36	1.398 (5)
C6—H6	0.9500	C32—C33	1.391 (5)
C7—C8	1.375 (6)	C32—H32	0.9500
C7—H7	0.9500	C33—C34	1.379 (5)
C8—C9	1.376 (7)	C33—H33	0.9500
C8—H8	0.9500	C34—C35	1.380 (6)
C9—C10	1.383 (6)	C34—H34	0.9500
C9—H9	0.9500	C35—C36	1.392 (5)
C10—H10	0.9500	C35—H35	0.9500
C11—C12	1.397 (5)	C36—H36	0.9500
C11—C16	1.393 (5)		
			
C21—Sn—C11	119.27 (13)	C12—C11—C16	118.2 (3)
C21—Sn—C31	105.46 (13)	C12—C11—Sn	117.4 (3)
C11—Sn—C31	106.56 (13)	C16—C11—Sn	124.4 (3)
C21—Sn—S1	117.16 (9)	C11—C12—C13	120.7 (3)
C11—Sn—S1	111.30 (9)	C11—C12—H12	119.7
C31—Sn—S1	92.68 (9)	C13—C12—H12	119.7
C21—Sn—S2	82.51 (9)	C14—C13—C12	120.2 (4)
C11—Sn—S2	88.60 (10)	C14—C13—H13	119.9
C31—Sn—S2	155.43 (9)	C12—C13—H13	119.9
S1—Sn—S2	63.42 (3)	C15—C14—C13	119.6 (3)
C1—S1—Sn	97.50 (13)	C15—C14—H14	120.2
C1—S2—Sn	78.01 (12)	C13—C14—H14	120.2
C1—N1—C2	122.8 (3)	C14—C15—C16	120.5 (4)
C1—N1—C3	121.4 (3)	C14—C15—H15	119.7
C2—N1—C3	115.8 (3)	C16—C15—H15	119.7
N1—C1—S2	124.1 (3)	C15—C16—C11	120.7 (4)
N1—C1—S1	115.9 (3)	C15—C16—H16	119.6
S2—C1—S1	120.0 (2)	C11—C16—H16	119.6
N1—C2—H2A	109.5	C22—C21—C26	118.2 (3)
N1—C2—H2B	109.5	C22—C21—Sn	120.9 (3)
H2A—C2—H2B	109.5	C26—C21—Sn	120.8 (3)
N1—C2—H2C	109.5	C23—C22—C21	121.1 (4)
H2A—C2—H2C	109.5	C23—C22—H22	119.4
H2B—C2—H2C	109.5	C21—C22—H22	119.4
N1—C3—C4	111.5 (3)	C24—C23—C22	119.9 (4)
N1—C3—H3A	109.3	C24—C23—H23	120.1
C4—C3—H3A	109.3	C22—C23—H23	120.1
N1—C3—H3B	109.3	C23—C24—C25	120.0 (4)
C4—C3—H3B	109.3	C23—C24—H24	120.0
H3A—C3—H3B	108.0	C25—C24—H24	120.0
C5—C4—C3	111.5 (3)	C24—C25—C26	120.6 (4)
C5—C4—H4A	109.3	C24—C25—H25	119.7
C3—C4—H4A	109.3	C26—C25—H25	119.7
C5—C4—H4B	109.3	C25—C26—C21	120.3 (4)
C3—C4—H4B	109.3	C25—C26—H26	119.9
H4A—C4—H4B	108.0	C21—C26—H26	119.9
C6—C5—C10	118.0 (4)	C32—C31—C36	117.6 (3)
C6—C5—C4	120.8 (4)	C32—C31—Sn	121.1 (2)
C10—C5—C4	121.2 (4)	C36—C31—Sn	121.3 (3)
C7—C6—C5	120.9 (4)	C31—C32—C33	120.8 (3)
C7—C6—H6	119.6	C31—C32—H32	119.6
C5—C6—H6	119.6	C33—C32—H32	119.6
C6—C7—C8	121.0 (4)	C34—C33—C32	120.3 (4)
C6—C7—H7	119.5	C34—C33—H33	119.8
C8—C7—H7	119.5	C32—C33—H33	119.8
C7—C8—C9	118.8 (4)	C35—C34—C33	120.0 (3)
C7—C8—H8	120.6	C35—C34—H34	120.0
C9—C8—H8	120.6	C33—C34—H34	120.0
C8—C9—C10	120.8 (4)	C34—C35—C36	120.0 (3)
C8—C9—H9	119.6	C34—C35—H35	120.0
C10—C9—H9	119.6	C36—C35—H35	120.0
C9—C10—C5	120.5 (4)	C35—C36—C31	121.2 (3)
C9—C10—H10	119.7	C35—C36—H36	119.4
C5—C10—H10	119.7	C31—C36—H36	119.4
			
C2—N1—C1—S2	−177.8 (3)	C11—C12—C13—C14	−1.5 (6)
C3—N1—C1—S2	3.7 (5)	C12—C13—C14—C15	0.2 (6)
C2—N1—C1—S1	2.4 (5)	C13—C14—C15—C16	1.1 (6)
C3—N1—C1—S1	−176.0 (3)	C14—C15—C16—C11	−1.1 (6)
Sn—S2—C1—N1	171.3 (3)	C12—C11—C16—C15	−0.2 (5)
Sn—S2—C1—S1	−9.00 (18)	Sn—C11—C16—C15	179.9 (3)
Sn—S1—C1—N1	−169.0 (3)	C26—C21—C22—C23	−0.2 (6)
Sn—S1—C1—S2	11.2 (2)	Sn—C21—C22—C23	176.5 (3)
C1—N1—C3—C4	102.6 (4)	C21—C22—C23—C24	−0.3 (7)
C2—N1—C3—C4	−76.0 (4)	C22—C23—C24—C25	0.9 (7)
N1—C3—C4—C5	−175.8 (3)	C23—C24—C25—C26	−1.1 (7)
C3—C4—C5—C6	91.9 (5)	C24—C25—C26—C21	0.5 (7)
C3—C4—C5—C10	−86.7 (5)	C22—C21—C26—C25	0.1 (6)
C10—C5—C6—C7	0.4 (6)	Sn—C21—C26—C25	−176.6 (3)
C4—C5—C6—C7	−178.2 (4)	C36—C31—C32—C33	1.2 (5)
C5—C6—C7—C8	0.0 (7)	Sn—C31—C32—C33	179.6 (3)
C6—C7—C8—C9	−0.3 (7)	C31—C32—C33—C34	−0.9 (6)
C7—C8—C9—C10	0.2 (7)	C32—C33—C34—C35	−0.3 (6)
C8—C9—C10—C5	0.2 (8)	C33—C34—C35—C36	1.1 (6)
C6—C5—C10—C9	−0.5 (7)	C34—C35—C36—C31	−0.7 (6)
C4—C5—C10—C9	178.1 (4)	C32—C31—C36—C35	−0.4 (5)
C16—C11—C12—C13	1.5 (5)	Sn—C31—C36—C35	−178.8 (3)
Sn—C11—C12—C13	−178.6 (3)		

### [N-Methyl-N-(2-phenylethyl)dithiocarbamato-κ2S,S']triphenyltin(IV) (II) Hydrogen-bond geometry (Å, º)

Hydrogen-bond geometry (Å, °) for (I). Cg1 and Cg2 are the ring centroids of the (C11–C16) and (C31–C36) rings, respectively.

**Table:**

*D*—H···*A*	*D*—H	H···*A*	*D*···*A*	*D*—H···*A*
C2—H2*B*···*Cg*1^i^	0.98	2.99	3.779 (5)	138
C14—H14···*Cg*2^ii^	0.95	2.95	3.754 (5)	143

Symmetry codes: (i) −*x*+2, −*y*, −*z*+2; (ii) *x*, *y*−1, *z*.
